# 5-Cyclo­hexyl-3-cyclo­hexyl­sulfonyl-2-methyl-1-benzofuran

**DOI:** 10.1107/S1600536811007872

**Published:** 2011-03-09

**Authors:** Hong Dae Choi, Pil Ja Seo, Byeng Wha Son, Uk Lee

**Affiliations:** a Department of Chemistry, Dongeui University, San 24 Kaya-dong Busanjin-gu, Busan 614-714, Republic of Korea; b Department of Chemistry, Pukyong National University, 599-1 Daeyeon 3-dong, Nam-gu, Busan 608-737, Republic of Korea

## Abstract

In the title compound, C_21_H_28_O_3_S, the benzofuran unit is essentially planar, with a mean deviation of 0.016 (1) Å from the least-squares plane defined by the nine constituent atoms. Both cyclo­hexane rings adopt chair conformations.

## Related literature

For the pharmacological activity of benzofuran compounds, see: Aslam *et al.* (2006 ▶); Galal *et al.* (2009 ▶); Khan *et al.* (2005 ▶). For natural products with benzofuran rings, see: Akgul & Anil (2003 ▶); Soekamto *et al.* (2003 ▶). For structural studies of related 2-aryl-5-cyclo­hexyl-3-methyl­sufinyl-1-benzofuran derivatives, see: Choi *et al.* (2011*a* ▶,*b* ▶).
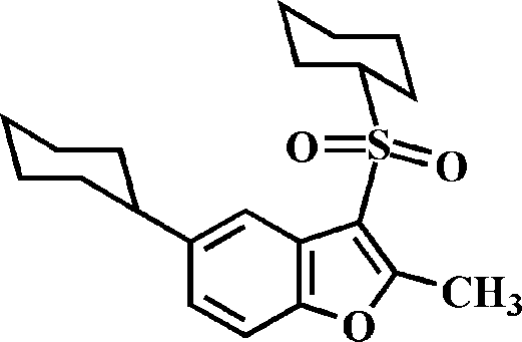


## Experimental

### 

#### Crystal data

C_21_H_28_O_3_S
*M* *_r_* = 360.49
Triclinic, 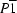

*a* = 6.8819 (2) Å
*b* = 10.7244 (3) Å
*c* = 13.3533 (4) Å
α = 77.132 (2)°
β = 83.165 (2)°
γ = 87.597 (2)°
*V* = 953.83 (5) Å^3^
*Z* = 2
Mo *K*α radiation
μ = 0.19 mm^−1^
*T* = 298 K
0.20 × 0.17 × 0.17 mm

#### Data collection

Bruker SMART APEXII CCD diffractometer
Absorption correction: multi-scan (*SADABS*; Bruker, 2009 ▶) *T*_min_ = 0.963, *T*_max_ = 0.969
17113 measured reflections
4397 independent reflections
3140 reflections with *I* > 2σ(*I*)
*R*_int_ = 0.032

#### Refinement

*R*[*F* ^2^ > 2σ(*F* ^2^)] = 0.048
*wR*(*F* ^2^) = 0.136
*S* = 1.06
4397 reflections
227 parameters
H-atom parameters constrained
Δρ_max_ = 0.17 e Å^−3^
Δρ_min_ = −0.32 e Å^−3^

### 

Data collection: *APEX2* (Bruker, 2009 ▶); cell refinement: *SAINT* (Bruker, 2009 ▶); data reduction: *SAINT*; program(s) used to solve structure: *SHELXS97* (Sheldrick, 2008 ▶); program(s) used to refine structure: *SHELXL97* (Sheldrick, 2008 ▶); molecular graphics: *ORTEP-3* (Farrugia, 1997 ▶); software used to prepare material for publication: *SHELXL97*.

## Supplementary Material

Crystal structure: contains datablocks I. DOI: 10.1107/S1600536811007872/nk2090sup1.cif

Structure factors: contains datablocks I. DOI: 10.1107/S1600536811007872/nk2090Isup2.hkl

Additional supplementary materials:  crystallographic information; 3D view; checkCIF report

## Figures and Tables

**Table 1:** Hydrogen-bond geometry (Å, °) *Cg* is the centroid of the C1/C2/C7/O1/C8 furan ring.

*D*—H⋯*A*	*D*—H	H⋯*A*	*D*⋯*A*	*D*—H⋯*A*
C14—H14*B*⋯*Cg*^i^	0.97	2.80	3.649 (3)	147

Symmetry code: (i) 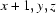.

**Fig. 1.**
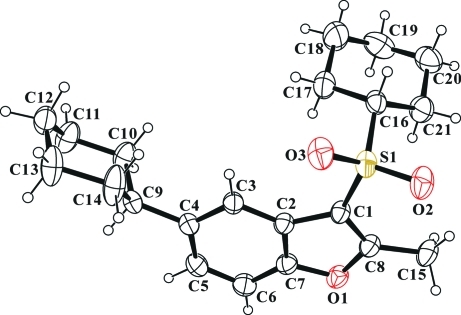
The molecular structure of the title compound. Displacement ellipsoids are drawn at the 30% probability level. H atoms are presented as a small spheres of arbitrary radius.

## supplementary crystallographic information

### Comment

Many compounds involving a benzofuran ring have attracted interesting pharmacological properties such as antifungal, antitumor and antiviral, and antimicrobial activities (Aslam *et al.*, 2006, Galal *et al.*, 2009, Khan *et al.*, 2005). These compounds occur in a wide range of natural products (Akgul & Anil, 2003; Soekamto *et al.*, 2003). As a part of our ongoing study of the substituent effect on the solid state structures of 2-aryl-5-cyclohexyl-3-methylsufinyl-1-benzofuran analogues (Choi *et al.*, 2011**a*, b*), we report herein on the crystal structure of the title compound.

In the title molecule (Fig. 1), the benzofuran unit is essentially planar, with a mean deviation of 0.016 (1) Å from the least-squares plane defined by the nine constituent atoms. The cyclohexane rings in each are in the chair form.

The molecular packing is stabilized by weak intermolecular C—H···π interactions between a cyclohexyl H atom in the 5-position and the furan ring (Table 1).

### Experimental

77% 3-chloroperoxybenzoic acid (448 mg, 2.0 mmol) was added in small portions to a stirred solution of 5-cyclohexyl-3-cyclohexylsulfanyl-2-methyl-1-benzofuran (295 mg, 0.9 mmol) in dichloromethane (40 mL) at 273 K. After being stirred at room temperature for 6h, the mixture was washed with saturated sodium bicarbonate solution and the organic layer was separated, dried over magnesium sulfate, filtered and concentrated at reduced pressure. The residue was purified by column chromatography (benzene) to afford the title compound as a colorless solid [yield 79%, m.p. 378–379 K; *R*_f_ = 0.65 (benzene)]. Single crystals suitable for X-ray diffraction were prepared by slow evaporation of a solution of the title compound in ethyl acetate at room temperature.

### Refinement

All H atoms were positioned geometrically and refined using a riding model, with C—H = 0.95 Å for aryl, 1.00 Å for methine, 0.99 Å for methylene and 0.98 Å for methyl H atoms, respectively. *U*_iso_(H) = 1.2*U*_eq_(C) for aryl, methine and methylene, and 1.5*U*_eq_(C) for methyl H atoms.

### Crystal data

**Table:**

C_21_H_28_O_3_S	*Z* = 2
*M**_r_* = 360.49	*F*(000) = 388
Triclinic, *P*1	*D*_x_ = 1.255 Mg m^−^^3^
Hall symbol: -P 1	Mo *K*α radiation, λ = 0.71073 Å
*a* = 6.8819 (2) Å	Cell parameters from 6431 reflections
*b* = 10.7244 (3) Å	θ = 2.6–27.5°
*c* = 13.3533 (4) Å	µ = 0.19 mm^−^^1^
α = 77.132 (2)°	*T* = 298 K
β = 83.165 (2)°	Block, colourless
γ = 87.597 (2)°	0.20 × 0.17 × 0.17 mm
*V* = 953.83 (5) Å^3^	

### Data collection

**Table:**

Bruker SMART APEXII CCD diffractometer	4397 independent reflections
Radiation source: rotating anode	3140 reflections with *I* > 2σ(*I*)
graphite multilayer	*R*_int_ = 0.032
Detector resolution: 10.0 pixels mm^-1^	θ_max_ = 27.6°, θ_min_ = 1.6°
φ and ω scans	*h* = −8→8
Absorption correction: multi-scan (*SADABS*; Bruker, 2009)	*k* = −13→13
*T*_min_ = 0.963, *T*_max_ = 0.969	*l* = −17→17
17113 measured reflections	

### Refinement

**Table:**

Refinement on *F*^2^	Primary atom site location: structure-invariant direct methods
Least-squares matrix: full	Secondary atom site location: difference Fourier map
*R*[*F*^2^ > 2σ(*F*^2^)] = 0.048	Hydrogen site location: difference Fourier map
*wR*(*F*^2^) = 0.136	H-atom parameters constrained
*S* = 1.06	*w* = 1/[σ^2^(*F*_o_^2^) + (0.0644*P*)^2^ + 0.1575*P*] where *P* = (*F*_o_^2^ + 2*F*_c_^2^)/3
4397 reflections	(Δ/σ)_max_ < 0.001
227 parameters	Δρ_max_ = 0.17 e Å^−^^3^
0 restraints	Δρ_min_ = −0.32 e Å^−^^3^

### Special details

**Table:**

Geometry. All esds (except the esd in the dihedral angle between two l.s. planes) are estimated using the full covariance matrix. The cell esds are taken into account individually in the estimation of esds in distances, angles and torsion angles; correlations between esds in cell parameters are only used when they are defined by crystal symmetry. An approximate (isotropic) treatment of cell esds is used for estimating esds involving l.s. planes.
Refinement. Refinement of F^2^ against ALL reflections. The weighted R-factor wR and goodness of fit S are based on F^2^, conventional R-factors R are based on F, with F set to zero for negative F^2^. The threshold expression of F^2^ > 2sigma(F^2^) is used only for calculating R-factors(gt) etc. and is not relevant to the choice of reflections for refinement. R-factors based on F^2^ are statistically about twice as large as those based on F, and R- factors based on ALL data will be even larger.

### Fractional atomic coordinates and isotropic or equivalent isotropic displacement parameters (Å^2^)

**Table:**

	*x*	*y*	*z*	*U*_iso_*/*U*_eq_	
S1	0.52844 (7)	0.02769 (5)	0.68510 (4)	0.05492 (17)	
O1	0.24896 (19)	0.06179 (12)	0.95160 (10)	0.0577 (3)	
O2	0.4736 (2)	−0.10029 (13)	0.68671 (12)	0.0741 (4)	
O3	0.73307 (19)	0.05695 (15)	0.67114 (12)	0.0711 (4)	
C1	0.4324 (2)	0.06712 (16)	0.80074 (14)	0.0471 (4)	
C2	0.5173 (2)	0.15467 (16)	0.85153 (13)	0.0464 (4)	
C3	0.6739 (3)	0.23846 (17)	0.82839 (14)	0.0495 (4)	
H3	0.7569	0.2446	0.7673	0.059*	
C4	0.7042 (3)	0.31244 (17)	0.89796 (14)	0.0519 (4)	
C5	0.5794 (3)	0.2984 (2)	0.99068 (15)	0.0620 (5)	
H5	0.6019	0.3475	1.0373	0.074*	
C6	0.4256 (3)	0.2156 (2)	1.01573 (15)	0.0628 (5)	
H6	0.3448	0.2070	1.0778	0.075*	
C7	0.3978 (3)	0.14608 (17)	0.94388 (14)	0.0516 (4)	
C8	0.2723 (3)	0.01559 (17)	0.86374 (15)	0.0524 (4)	
C9	0.8639 (3)	0.41041 (18)	0.87500 (15)	0.0553 (5)	
H9	0.8911	0.4251	0.9418	0.066*	
C10	0.7991 (3)	0.53748 (19)	0.8132 (2)	0.0713 (6)	
H10A	0.6801	0.5662	0.8487	0.086*	
H10B	0.7702	0.5272	0.7462	0.086*	
C11	0.9558 (3)	0.6382 (2)	0.7982 (2)	0.0843 (7)	
H11A	0.9130	0.7165	0.7540	0.101*	
H11B	0.9718	0.6566	0.8646	0.101*	
C12	1.1487 (3)	0.5964 (2)	0.75125 (19)	0.0759 (6)	
H12A	1.1385	0.5911	0.6805	0.091*	
H12B	1.2466	0.6594	0.7500	0.091*	
C13	1.2116 (3)	0.4695 (2)	0.8108 (3)	0.0896 (8)	
H13A	1.2398	0.4778	0.8785	0.108*	
H13B	1.3307	0.4413	0.7752	0.108*	
C14	1.0539 (3)	0.3696 (2)	0.8236 (3)	0.0909 (9)	
H14A	1.0340	0.3558	0.7562	0.109*	
H14B	1.0975	0.2892	0.8646	0.109*	
C15	0.1213 (3)	−0.0750 (2)	0.85573 (18)	0.0670 (6)	
H15A	0.0979	−0.1363	0.9201	0.100*	
H15B	0.1659	−0.1187	0.8016	0.100*	
H15C	0.0021	−0.0288	0.8402	0.100*	
C16	0.4147 (3)	0.13219 (19)	0.58508 (15)	0.0563 (5)	
H16	0.4792	0.1166	0.5194	0.068*	
C17	0.4450 (4)	0.2720 (2)	0.5837 (2)	0.0849 (7)	
H17A	0.5839	0.2895	0.5741	0.102*	
H17B	0.3902	0.2906	0.6492	0.102*	
C18	0.3455 (6)	0.3575 (3)	0.4957 (3)	0.1165 (12)	
H18A	0.3574	0.4463	0.4988	0.140*	
H18B	0.4119	0.3462	0.4300	0.140*	
C19	0.1343 (5)	0.3274 (3)	0.5015 (2)	0.1029 (9)	
H19A	0.0784	0.3813	0.4431	0.124*	
H19B	0.0652	0.3460	0.5643	0.124*	
C20	0.1080 (4)	0.1899 (3)	0.5006 (2)	0.0874 (8)	
H20A	0.1686	0.1729	0.4355	0.105*	
H20B	−0.0305	0.1724	0.5064	0.105*	
C21	0.1990 (3)	0.1027 (2)	0.58921 (18)	0.0741 (6)	
H21A	0.1858	0.0142	0.5852	0.089*	
H21B	0.1303	0.1140	0.6544	0.089*	

### Atomic displacement parameters (Å^2^)

**Table:**

	*U*^11^	*U*^22^	*U*^33^	*U*^12^	*U*^13^	*U*^23^
S1	0.0445 (3)	0.0590 (3)	0.0664 (3)	−0.00717 (19)	0.0051 (2)	−0.0295 (2)
O1	0.0555 (8)	0.0588 (8)	0.0563 (8)	−0.0121 (6)	0.0084 (6)	−0.0129 (6)
O2	0.0773 (10)	0.0585 (8)	0.0948 (11)	−0.0075 (7)	0.0032 (8)	−0.0399 (8)
O3	0.0423 (7)	0.0937 (11)	0.0834 (10)	−0.0075 (7)	0.0080 (7)	−0.0388 (8)
C1	0.0429 (9)	0.0460 (9)	0.0532 (10)	−0.0072 (7)	0.0013 (7)	−0.0146 (7)
C2	0.0449 (9)	0.0464 (9)	0.0482 (10)	−0.0030 (7)	−0.0029 (7)	−0.0118 (7)
C3	0.0474 (9)	0.0535 (10)	0.0489 (10)	−0.0065 (8)	−0.0039 (8)	−0.0141 (8)
C4	0.0538 (10)	0.0533 (10)	0.0513 (11)	−0.0043 (8)	−0.0124 (8)	−0.0130 (8)
C5	0.0721 (13)	0.0672 (12)	0.0534 (11)	−0.0031 (10)	−0.0104 (10)	−0.0253 (9)
C6	0.0672 (13)	0.0720 (13)	0.0501 (11)	−0.0050 (10)	0.0028 (9)	−0.0197 (9)
C7	0.0511 (10)	0.0531 (10)	0.0495 (10)	−0.0044 (8)	−0.0009 (8)	−0.0106 (8)
C8	0.0511 (10)	0.0488 (9)	0.0561 (11)	−0.0067 (8)	0.0005 (8)	−0.0115 (8)
C9	0.0615 (11)	0.0590 (11)	0.0520 (11)	−0.0098 (9)	−0.0159 (9)	−0.0196 (9)
C10	0.0525 (12)	0.0555 (11)	0.1044 (18)	−0.0008 (9)	−0.0057 (11)	−0.0160 (11)
C11	0.0698 (15)	0.0536 (12)	0.126 (2)	−0.0079 (11)	−0.0088 (14)	−0.0119 (12)
C12	0.0646 (13)	0.0882 (16)	0.0786 (15)	−0.0252 (12)	−0.0015 (11)	−0.0255 (12)
C13	0.0465 (12)	0.0799 (16)	0.156 (3)	−0.0051 (11)	−0.0167 (14)	−0.0507 (16)
C14	0.0462 (12)	0.0634 (13)	0.173 (3)	0.0006 (10)	−0.0215 (14)	−0.0417 (15)
C15	0.0567 (12)	0.0609 (12)	0.0811 (15)	−0.0215 (9)	0.0072 (10)	−0.0147 (10)
C16	0.0526 (10)	0.0691 (12)	0.0516 (11)	−0.0153 (9)	0.0048 (8)	−0.0257 (9)
C17	0.1043 (19)	0.0656 (14)	0.0884 (18)	−0.0248 (13)	−0.0321 (15)	−0.0093 (12)
C18	0.159 (3)	0.0811 (18)	0.108 (2)	−0.0358 (19)	−0.062 (2)	0.0120 (16)
C19	0.120 (3)	0.116 (2)	0.0763 (18)	0.0200 (19)	−0.0360 (17)	−0.0212 (16)
C20	0.0658 (14)	0.125 (2)	0.0737 (16)	−0.0128 (14)	−0.0137 (12)	−0.0223 (15)
C21	0.0550 (12)	0.0915 (16)	0.0764 (15)	−0.0170 (11)	−0.0042 (11)	−0.0182 (12)

### Geometric parameters (Å, °)

**Table:**

S1—O2	1.4336 (14)	C12—C13	1.491 (4)
S1—O3	1.4370 (14)	C12—H12A	0.9700
S1—C1	1.7393 (17)	C12—H12B	0.9700
S1—C16	1.778 (2)	C13—C14	1.525 (3)
O1—C8	1.362 (2)	C13—H13A	0.9700
O1—C7	1.374 (2)	C13—H13B	0.9700
C1—C8	1.360 (2)	C14—H14A	0.9700
C1—C2	1.452 (2)	C14—H14B	0.9700
C2—C7	1.385 (2)	C15—H15A	0.9600
C2—C3	1.394 (2)	C15—H15B	0.9600
C3—C4	1.387 (2)	C15—H15C	0.9600
C3—H3	0.9300	C16—C17	1.518 (3)
C4—C5	1.403 (3)	C16—C21	1.523 (3)
C4—C9	1.510 (2)	C16—H16	0.9800
C5—C6	1.372 (3)	C17—C18	1.532 (4)
C5—H5	0.9300	C17—H17A	0.9700
C6—C7	1.374 (3)	C17—H17B	0.9700
C6—H6	0.9300	C18—C19	1.491 (4)
C8—C15	1.481 (3)	C18—H18A	0.9700
C9—C14	1.498 (3)	C18—H18B	0.9700
C9—C10	1.507 (3)	C19—C20	1.496 (4)
C9—H9	0.9800	C19—H19A	0.9700
C10—C11	1.523 (3)	C19—H19B	0.9700
C10—H10A	0.9700	C20—C21	1.517 (3)
C10—H10B	0.9700	C20—H20A	0.9700
C11—C12	1.495 (3)	C20—H20B	0.9700
C11—H11A	0.9700	C21—H21A	0.9700
C11—H11B	0.9700	C21—H21B	0.9700
			
O2—S1—O3	118.46 (9)	C12—C13—C14	111.4 (2)
O2—S1—C1	108.89 (9)	C12—C13—H13A	109.3
O3—S1—C1	106.41 (8)	C14—C13—H13A	109.3
O2—S1—C16	107.65 (9)	C12—C13—H13B	109.3
O3—S1—C16	108.06 (9)	C14—C13—H13B	109.3
C1—S1—C16	106.83 (9)	H13A—C13—H13B	108.0
C8—O1—C7	107.00 (13)	C9—C14—C13	111.67 (19)
C8—C1—C2	107.30 (15)	C9—C14—H14A	109.3
C8—C1—S1	126.59 (14)	C13—C14—H14A	109.3
C2—C1—S1	125.98 (13)	C9—C14—H14B	109.3
C7—C2—C3	119.25 (16)	C13—C14—H14B	109.3
C7—C2—C1	104.34 (15)	H14A—C14—H14B	107.9
C3—C2—C1	136.40 (16)	C8—C15—H15A	109.5
C4—C3—C2	118.98 (17)	C8—C15—H15B	109.5
C4—C3—H3	120.5	H15A—C15—H15B	109.5
C2—C3—H3	120.5	C8—C15—H15C	109.5
C3—C4—C5	119.08 (18)	H15A—C15—H15C	109.5
C3—C4—C9	121.70 (17)	H15B—C15—H15C	109.5
C5—C4—C9	119.20 (16)	C17—C16—C21	111.8 (2)
C6—C5—C4	123.02 (18)	C17—C16—S1	112.23 (14)
C6—C5—H5	118.5	C21—C16—S1	111.96 (14)
C4—C5—H5	118.5	C17—C16—H16	106.8
C5—C6—C7	116.15 (18)	C21—C16—H16	106.8
C5—C6—H6	121.9	S1—C16—H16	106.8
C7—C6—H6	121.9	C16—C17—C18	109.98 (19)
C6—C7—O1	125.66 (17)	C16—C17—H17A	109.7
C6—C7—C2	123.50 (18)	C18—C17—H17A	109.7
O1—C7—C2	110.83 (15)	C16—C17—H17B	109.7
C1—C8—O1	110.53 (16)	C18—C17—H17B	109.7
C1—C8—C15	134.11 (18)	H17A—C17—H17B	108.2
O1—C8—C15	115.35 (16)	C19—C18—C17	112.1 (2)
C14—C9—C10	109.39 (18)	C19—C18—H18A	109.2
C14—C9—C4	115.03 (16)	C17—C18—H18A	109.2
C10—C9—C4	112.23 (16)	C19—C18—H18B	109.2
C14—C9—H9	106.5	C17—C18—H18B	109.2
C10—C9—H9	106.5	H18A—C18—H18B	107.9
C4—C9—H9	106.5	C18—C19—C20	111.2 (3)
C9—C10—C11	111.57 (18)	C18—C19—H19A	109.4
C9—C10—H10A	109.3	C20—C19—H19A	109.4
C11—C10—H10A	109.3	C18—C19—H19B	109.4
C9—C10—H10B	109.3	C20—C19—H19B	109.4
C11—C10—H10B	109.3	H19A—C19—H19B	108.0
H10A—C10—H10B	108.0	C19—C20—C21	110.9 (2)
C12—C11—C10	112.5 (2)	C19—C20—H20A	109.5
C12—C11—H11A	109.1	C21—C20—H20A	109.5
C10—C11—H11A	109.1	C19—C20—H20B	109.5
C12—C11—H11B	109.1	C21—C20—H20B	109.5
C10—C11—H11B	109.1	H20A—C20—H20B	108.1
H11A—C11—H11B	107.8	C20—C21—C16	110.72 (18)
C13—C12—C11	111.2 (2)	C20—C21—H21A	109.5
C13—C12—H12A	109.4	C16—C21—H21A	109.5
C11—C12—H12A	109.4	C20—C21—H21B	109.5
C13—C12—H12B	109.4	C16—C21—H21B	109.5
C11—C12—H12B	109.4	H21A—C21—H21B	108.1
H12A—C12—H12B	108.0		
			
O2—S1—C1—C8	−25.2 (2)	C7—O1—C8—C1	0.5 (2)
O3—S1—C1—C8	−153.94 (17)	C7—O1—C8—C15	−178.31 (16)
C16—S1—C1—C8	90.80 (18)	C3—C4—C9—C14	−40.4 (3)
O2—S1—C1—C2	149.95 (16)	C5—C4—C9—C14	141.5 (2)
O3—S1—C1—C2	21.20 (18)	C3—C4—C9—C10	85.5 (2)
C16—S1—C1—C2	−94.06 (17)	C5—C4—C9—C10	−92.6 (2)
C8—C1—C2—C7	0.7 (2)	C14—C9—C10—C11	−55.7 (3)
S1—C1—C2—C7	−175.17 (14)	C4—C9—C10—C11	175.38 (19)
C8—C1—C2—C3	−177.6 (2)	C9—C10—C11—C12	54.6 (3)
S1—C1—C2—C3	6.4 (3)	C10—C11—C12—C13	−53.1 (3)
C7—C2—C3—C4	−0.9 (3)	C11—C12—C13—C14	53.8 (3)
C1—C2—C3—C4	177.26 (19)	C10—C9—C14—C13	57.1 (3)
C2—C3—C4—C5	1.6 (3)	C4—C9—C14—C13	−175.5 (2)
C2—C3—C4—C9	−176.48 (16)	C12—C13—C14—C9	−57.0 (3)
C3—C4—C5—C6	−0.9 (3)	O2—S1—C16—C17	173.88 (16)
C9—C4—C5—C6	177.21 (19)	O3—S1—C16—C17	−57.08 (18)
C4—C5—C6—C7	−0.5 (3)	C1—S1—C16—C17	57.06 (17)
C5—C6—C7—O1	−177.38 (18)	O2—S1—C16—C21	47.25 (17)
C5—C6—C7—C2	1.2 (3)	O3—S1—C16—C21	176.29 (14)
C8—O1—C7—C6	178.74 (19)	C1—S1—C16—C21	−69.57 (16)
C8—O1—C7—C2	0.0 (2)	C21—C16—C17—C18	−53.7 (3)
C3—C2—C7—C6	−0.5 (3)	S1—C16—C17—C18	179.6 (2)
C1—C2—C7—C6	−179.22 (19)	C16—C17—C18—C19	54.7 (4)
C3—C2—C7—O1	178.26 (15)	C17—C18—C19—C20	−57.0 (3)
C1—C2—C7—O1	−0.5 (2)	C18—C19—C20—C21	57.5 (3)
C2—C1—C8—O1	−0.8 (2)	C19—C20—C21—C16	−56.4 (3)
S1—C1—C8—O1	175.11 (13)	C17—C16—C21—C20	55.3 (2)
C2—C1—C8—C15	177.7 (2)	S1—C16—C21—C20	−177.83 (17)
S1—C1—C8—C15	−6.4 (3)		

### Hydrogen-bond geometry (Å, °)

**Table:**

Cg is the centroid of the C1/C2/C7/O1/C8 furan ring.

**Table:**

*D*—H···*A*	*D*—H	H···*A*	*D*···*A*	*D*—H···*A*
C14—H14B···Cg^i^	0.97	2.80	3.649 (3)	147

Symmetry codes: (i) *x*+1, *y*, *z*.
